# Knockout cancer by nano-delivered immunotherapy using perfusion-aided scaffold-based tumor-on-a-chip

**DOI:** 10.7150/ntno.87818

**Published:** 2024-03-31

**Authors:** Pooja Suryavanshi, Dhananjay Bodas

**Affiliations:** 1 Nanobioscience Group, Agharkar Research Institute, G.G. Agarkar Road, Pune 411 004 India.; 2 Savitribai Phule Pune University, Ganeshkhind Road, Pune 411 007 India.

**Keywords:** 3D cell culture, microfluidics, perfusion, multicellular tumor, *in vitro* immunotherapy

## Abstract

Cancer is a multifactorial disease produced by mutations in the oncogenes and tumor suppressor genes, which result in uncontrolled cell proliferation and resistance to cell death. Cancer progresses due to the escape of altered cells from immune monitoring, which is facilitated by the tumor's mutual interaction with its microenvironment. Understanding the mechanisms involved in immune surveillance evasion and the significance of the tumor microenvironment might thus aid in developing improved therapies. Although in vivo models are commonly utilized, they could be better for time, cost, and ethical concerns. As a result, it is critical to replicate an in vivo model and recreate the cellular and tissue-level functionalities. A 3D cell culture, which gives a 3D architecture similar to that found in vivo, is an appropriate model.

Furthermore, numerous cell types can be cocultured, establishing cellular interactions between TME and tumor cells. Moreover, microfluidics perfusion can provide precision flow rates, thus simulating tissue/organ function. Immunotherapy can be used with the perfused 3D cell culture technique to help develop successful therapeutics. Immunotherapy employing nano delivery can target the spot and silence the responsible genes, ensuring treatment effectiveness while minimizing adverse effects. This study focuses on the importance of 3D cell culture in understanding the pathophysiology of 3D tumors and TME, the function of TME in drug resistance, tumor progression, and the development of advanced anticancer therapies for high-throughput drug screening.

## 1. Introduction

Cancer is the primary cause of death worldwide, decreasing life expectancy. 1 The cancer incidence report shows 19.3 million new cases, with 10 million deaths worldwide, with the burden anticipated to rise to 28.4 million by 2040. 2 It occurs due to the transformation of a normal cell into tumor cells through altered genetic and epigenetic changes 3 and the rearrangement in the tumor microenvironment (TME). 5 During tumor progression, cells turn malignant due to self-sufficiency in growth signals, insensitivity to inhibitory growth signals, evasion of programmed cell death, limitless replicative potential, sustained angiogenesis, tissue invasion, and metastasis. Moreover, the interaction of ECM with tumor cells influences tumorigenesis by clonal evolution, cancer heterogeneity, epithelial-mesenchymal transition, migration, invasion, metastasis, and drug resistance. 5,6 Furthermore, the tumor cells establish the communication link with the TME and control the surrounding stromal cells for tumor proliferation and growth.

Therefore, different anticancer treatments, such as nucleoside analogs, taxanes, etc., have been primarily used for cancer treatment. However, these drugs suffer from low bioavailability, high-dose necessity, harmful side effects, low therapeutic indices, multidrug resistance, and non-specific targeting. 7 It causes direct or indirect damage to the kidney, affects the patient's hearing ability, and impairs the peripheral sensory neurons. In addition, it affects blood cell production and reduces liver function. 8 Besides these physiological side effects, 54% of cancer patients experience depression, anxiety, fear, and uncertainty about the cure. Moreover, 49% of cancer survivors suffer from fear of cancer recurrence (FCR) after chemotherapeutic treatment. 9

Recently, targeted therapies such as molecular therapy, antiangiogenesis therapy, apoptosis regulation, and signal transduction have been used for cancer treatment. Molecular therapy interferes with the specific biochemical pathways in tumor growth and development, while anti-antiangiogenesis therapy targets endothelial cells and inhibits new blood vessel formation. 10 Apoptosis regulation is based on the inhibition of tumors by targeting apoptotic inhibitors for apoptosis induction. 11 Signal-transduction therapy targets signaling elements responsible for tumor growth and survival. 12

Alternatively, gene therapy has been widely used for cancer treatment wherein a standard copy of the gene is inserted in the place of the defective gene to target the expression of pro-apoptotic genes, 13 chemo-sensitizing genes, and tumor suppressor genes. 14 Despite the success, there are still challenges to face while dealing with gene therapy, such as selecting the right conditions for optimal gene expression levels and the appropriate delivery system to target cancer cells. Some natural antioxidants such as vitamins, alkaloids, flavonoids, carotenoids, curcumin, berberine, and quercetin have been reported as anti-proliferative agents and introduced as complementary therapies for cancer. 15 Lately, cancer therapies such as hyperthermia with engineered nanomaterials in combination with other cancer drugs to target the tumor have also been used. 16 However, the detrimental side effects of chemotherapeutic drugs and tumor relapse (36 to 100%) warrant the development of reliable treatment with a sound understanding of the disease. 14

Cancer escapes the immune surveillance by producing neoantigens, adapting immune checkpoint footprints, and creating an immunosuppressive environment by establishing the crosstalk with surrounding TME. 24 TME comprises of tumor cells, endothelial cells, tumor-associated fibroblasts, and immune cells that play a significant role in tumor growth, metastasis, migration, and chemotherapeutic resistance. 18 To understand the role of TME in hijacking immune response and promoting tumor growth, developing a reliable model that recapitulates *in vivo*-like conditions is essential. The scaffold-based 3D culture structurally supports the formation of TME by coculture of tumor and stromal cells. 19 Moreover, 3D cell culture mimics the pathophysiology of *in vivo* solid tumors by providing cell-cell and cell-matrix interaction, signal transduction, cell differentiation, hypoxic core, and nutrient and oxygen gradient. 20 It increases the complexity of the tumor by concentric positioning of peripheral proliferating cells, viable intermediate cells, and necrotic core cells to create drug resistance that recapitulates the natural structure and function of *in vivo* solid tumors. 21

However, along with understanding the tumor pathophysiology in its native state, developing effective anticancer strategies is imperative. Naturally, the eradication of transformed cells is empowered by the native immune system that knocks out the disease and induces long-term immunity. 22 PD-1 and CTLA-4, T-cell surface receptors are responsible for tumor recognition and T-lymphocyte proliferation. Moreover, the binding of tumor neoantigen PDL-1 with PD-1 restricts the recognition of tumors and escapes immune surveillance along with several other processes. Therefore, T-cell surface receptors are modified to enhance tumor recognition and killing. 23 The chimeric antigen receptor (CAR-T) cell therapy uses engineering T-cell receptors to redirect the cytotoxic T-cells toward recognizing and eliminating tumors. 24 Lately, immune checkpoint blocked (ICB) therapy has been employed to downregulate the expression of PD-1 and CTLA-4 to restrict the binding of suppressor molecules secreted by a tumor in TME. These therapies could lead to the effective management of cancer. 25

The current review substantiates the role of 3D cell culture in understanding TME for developing and screening advanced anticancer therapies, that is, immunotherapy by the knockdown of T-cell surface receptors. The effect of ICB therapy can be enhanced by targeting nanoparticles formulated with chemotherapeutic drugs 26 to decrease the tumor burden. In conjunction, PD-1siRNA and CTLA-4 siRNA could be delivered to silence the gene expression to enhance tumor recognition, triggering an immune response against the tumor. 27, 28 The immunotherapeutic approach and 3D cell cultures will enable better understanding and high throughput drug screening to ensure zero-tolerance therapy towards cancer. It could also lead the way in developing a personalized regime to maximize patient compliance by reducing physiological and psychological side effects.

However, developing novel anticancer treatments demands understanding the mechanism of tumor generation and the role of the tumor microenvironment in tumor progression. Therefore, creating a tumor model that mimics the solid tumor's characteristic properties is essential. Cancer is a compact mass of tissue surrounded mainly by fibroblasts, immune cells, stem cells, and endothelial cells, creating heterogeneity along with a gradient of oxygen that contributes to the hypoxia and acidity in the tumor microenvironment that differentiates the tumor from normal metabolism. Thus, to understand these complexities, the tumor model should mimic the tumor properties such as heterogeneous tumor microenvironment, presence of cancer stem cells, oxygen gradient, cell energy variation, acidification of the cellular environment, and cell senescence.

## 2. The properties of solid tumors

3D cell culture techniques have been widely utilized that provide an invivo-like environment for studying tumor physiology and development, developing novel anticancer therapeutics, and high throughput drug screening.

3D cell culture plays a vital role in studying the behavior of solid tumors to investigate the effect of anticancer therapy, as a 2D culture fails to mimic *in vivo* solid tumors due to a lack of tumor microenvironment. Moreover, it helps in the concentric positioning of peripheral proliferating cells, viable intermediate cells, and necrotic core cells. Furthermore, enhanced interstitial pressure and physical barrier create drug resistance that recapitulates the natural structure and function of *in vivo* solid tumors. 21 The TME plays a vital role in tumor progression and metastasis and provides structural support where tumor cells receive biochemical signals from stromal cells. In addition, a gradient of oxygen, nutrients, metabolites, signaling molecules, and physical stress is present in solid tumors. Therefore, 3D cell culture provides an alternative with increased complexity created by multiple cell types in a single scaffold. 19

3D cell culture mimics *in vivo* solid tumors, thus aiding in research for drug discovery, drug screening, drug development, etc. Tumor spheroids mimic the characteristic features of solid tumors such as heterogeneous tumor microenvironment, presence of cancer stem cells, oxygen gradient, variation in cell energy, acidification of cellular environment, and cell senescence, making 3D spheroid an ideal technique for anticancer drug screening.

Tumor spheroids >500 μm in diameter established in a 3D cell culture platform can imitate different properties of solid tumors ranging in size from 0.5 to 1 mm^3^ through similar processes discovered in *in vivo* human solid tumors. These features impact the therapeutic efficacy of many drugs and other pharmacological compounds on spheroids. 29

### 2.1. Heterogenous tumor microenvironment

The TME consists of heterogeneous cellular composition, including tumor, stromal, immune, endothelial, and pericyte cells. Although these cells are non-transformed, they help in tumor progression by promoting angiogenesis, metastasis, proliferation, invasion, and drug resistance. The cells in TME interact with tumor cells by secretion of interleukins, fibroblast growth factors, insulin-like growth factors, etc., thus enhancing tumor cell proliferation. 30 Similarly, 3D tumor spheroids demonstrate TME by enabling the coculture of tumor and stromal cells and resistance mediated by tumor-stromal interaction. Lee et al. reported the coculture of cancerous and fibroblast cells. In addition, enhanced secretion of invasion markers such as TGF-β, N-cadherin and vimentin have also been reported. 31 Majeti et al. reported coculture of pancreatic, breast, and lung spheroid with fibroblast cells to enhance cell proliferation. Cells in coculture produce more epidermal growth factor (EGF), HGF, and IL6. The authors also discovered that increased expression of soluble factor contributed to cancer cell resistance, as pancreatic cancer cells in monoculture demonstrated approximately 50% survival to Erbitux treatment, whereas Bxpc3 cells in spheroids cocultured with fibroblasts demonstrated about 75% survival to the same treatment. 32 In addition, more than 90% of head‐and‐neck cancer cells, when cultured in 2D, died after 5 μM sorafenib or less than 10 μM cisplatin treatment. On the other hand, cells show 60% viability after 10 μM sorafenib or 20 μM cisplatin treatment when co-cultured with CAF cells. 33

Because stromal cells play a critical role in tumor resistance; several stromal-targeted therapies have been evaluated and reported. 34,35 Cancer-associated fibroblast (CAF) is a major cellular component of the tumor stroma; several therapeutics are being investigated to target fibroblast activation protein or their paracrine signaling pathways that is platelet-derived growth factor receptor, epithelial-mesenchymal transition (EMT), receptor tyrosine kinase and transforming growth factor (TGF) signaling pathways.

### 2.2. Oxygen gradient

Solid tumors display an oxygen gradient wherein the peripheral cells are exposed to more oxygen than the inner mass. 36 Grimes et al. reported the presence of an oxygen gradient in the tumor spheroids measured by the oxygen tension using an O_2_-sensitive microelectrode and fiber-optic oxygen sensor. 37 The cells on the outer side show higher proliferation than the inner core due to oxygen availability. Furthermore, spheroids show an upregulated expression of HIF-1α, indicating the presence of hypoxia in 3D cell culture. Tian et al. reported the higher expression of HIF‐1α protein in Hela tumor spheroids; however, the cells grown in monolayer did not show the HIF‐1α expression. 38 The expression of HIF-1α governs the resistance to many chemotherapeutic drugs by expressing P-glycoprotein on the surface of tumor cells, which actively exports the drug molecules outside the cell. Doublier et al. studied the contribution of high HIF-1α expression in the upregulation of P-glycoprotein, indicating the role of HIF-1α in cancer drug resistance. 39

In addition, the expression of VEGF is also observed in the tumor spheroids regulated by HIF-1α, contributing to drug resistance. 40 Gong et al. reported high expression of VEGF in the hypoxic core of the spheroid. 21 Qin et al. reported higher expression of VEGF in A375 melanoma spheroids compared to the cells cultured in 2D due to the metabolic variation in spheroidal cells responsible for the sensitivity of cancer cells towards vemurafenib. 41 Moreover, HIF-1α governs the fate of anti-apoptotic proteins such as Bak, Bax, Bcl‐xL, Bcl‐2, Bid, Mcl‐1 NF‐κB, and p53 in 3D spheroid. The hypoxic environment of 3D spheroid further induces drug resistance among the tumor cells. 42

Moreover, many researchers have already reported that the environment in the tumor spheroids supports drug resistance due to a hypoxic condition, as some therapeutics can induce the anticancer effect by generating ROS, which needs oxygen. 43 The hypoxic condition is also responsible for decreasing the damage mediated by radiation as the molecular oxygen reacts with ROS produced during the radiolysis of water, forming stable DNA peroxides that cause DNA damage. Khaitan et al. demonstrated that the BMG‐1 cell monolayers were more affected by the 0-10 Gy radiation than the spheroids. They have found the production of ROS in BMG‐1 spheroids after the radiation was higher than in the monolayer culture. 44

### 2.3. Variations in cell energy

**Figure 2:**
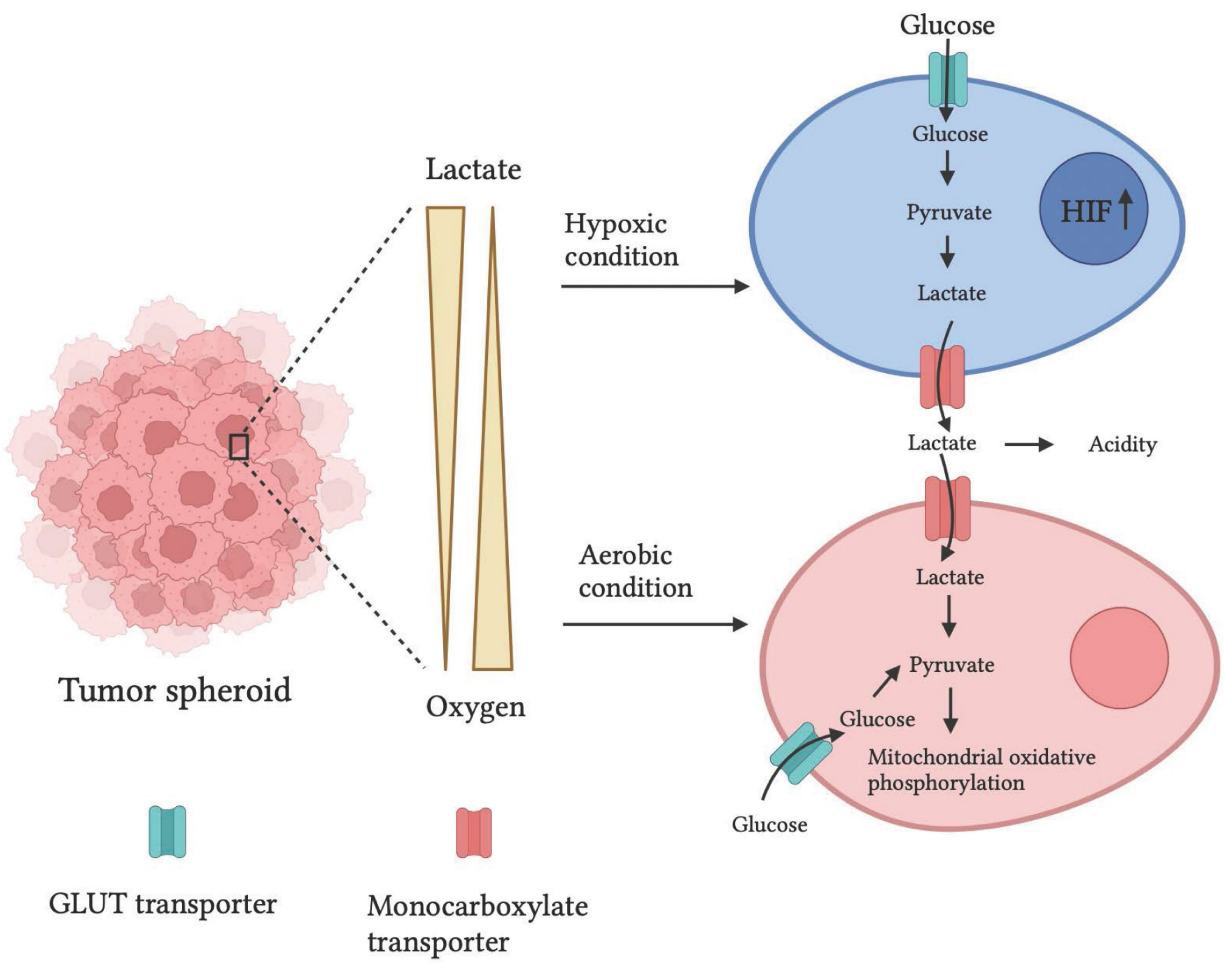
Representation of oxygen gradient and lactate availability in tumor spheroid that creates hypoxia and acidity in the tumor microenvironment and alters the metabolism of tumor cells. An illustration is adapted from ref. 50.

Normal human cells meet their energy demand from mitochondrial oxidative phosphorylation. However, due to the oxygen gradient and hypoxic conditions at the center, tumor cells utilize anaerobic respiration by producing lactate as a byproduct. There have been reports showing a similar mechanism in the 3D tumor spheroid. In addition, a high content of GLUT transport-1 and lactate dehydrogenase (Fig. 2) mRNA expression in 3D spheroids of PAN-1 cells compared to 2D cell cultures was observed. Moreover, increased GLUT-1 and lactate dehydrogenase expression create drug resistance in the spheroid. 45

Genetically encoded fluorescent biomarkers, PercevalHR and Phred, were developed to quantitatively assess ATP, ADP, and pH levels in MDA-MB-231 metastatic breast cancer cells and found a higher ATP: ADP ratio in denser cell matrices. 46 Pereira et al. discovered that GLUT1 expression in MCF7 breast cancer spheroids was 0.5-fold more significant than in monolayer cultures. 47 Furthermore, Khaitan et al. studied increased glucose consumption and lactate production two‐ to three-fold higher in BMG‐1 glioma spheroids than in BMG‐1 monolayer cell cultures. 44 It has been observed that increased GLUT1 and lactate generation due to the high glycolytic rate of cancer cells can contribute to drug resistance via altered expression of the multidrug resistance-associated protein 1 and P-glycoprotein. 48

Longati et al. discovered the mRNA expression ratios of glucose transporter 1 (GLUT1 is the predominant glucose transporter in many types of cancer) and lactate dehydrogenase (LDH; the enzyme responsible for lactate production) on PANC1 pancreatic cancer cells cultured in 2D/3D were approximately 3.5 and 7.5, respectively. 49

### 2.4. Acidification of the cellular environment

In solid tumors, lactate production is increased due to oxygen deficiency, which promotes acidification of the core. Similarly, acidification is observed in the tumor spheroid of CHT-29 colon carcinoma, U-251 Mg glioma, and HT-7 thyroid carcinoma with low pH in the center that interferes with the cellular uptake of an anticancer drug, resulting in drug resistance. 51 A pH-sensing electrode has been developed to generate high spatial and temporal resolution maps of pH gradients in paper-based cultures. These pH-sensing films will enable studies of extracellular gradients on cellular movement, viability, and drug resistance. 52 A macromolecular near-infrared poly ethylene glycol-conjugated iridium complex was developed to detect tumor acidity and hypoxia. The probe was used to detect primary tumors and metastatic tumor nodules in mice and to measure the metabolic rate of cancer cells* in vivo*. 53 Han et al. have developed an Iridium (III) based optical probe (Ir-1) that senses acidity and hypoxia in multicellular spheroids indicating acidic pH of 6.4 in the core than the outer circle. 54

The low pH levels reduce drug efficiency by interfering with cellular absorption. 55,56 In an acidic environment, some drugs, for example, doxorubicin, mitoxantrone, vincristine, vinblastine, anthraquinones, and vinca alkaloids, are protonated. As a result, the cellular uptake of these drugs is reduced because charged drugs are less effective in transposing cellular membranes. 57 Swietach et al. demonstrated the effect of spheroidal pH on drug absorption. These researchers discovered that doxorubicin absorption reduced proportionately to the inner core of the HCT116 colon cancer spheroids, indicating a higher IC50 value at pH 6.4. 58 In an acidic environment, the drug uptake efficiency is reduced because of low pH levels. 56,59

### 2.5. Cell senescence

The acidification of solid tumors due to lactate production decreases the availability of nutrients and oxygen, and thus, tumor cells enter the quiescent or senescence phase. However, they secrete the cytokines, chemokines, and growth factors that mediate tumor growth, progression, and drug resistance. 60

Gong et al. used flow cytometry to examine the cell cycle of MCF7 breast cancer cells cultivated in monolayers or spheroids. The results showed that 58.48 % of spheroidal cells were present in the quiescence or G0G1 phase of the cell cycle, compared to cells cultivated in 2D, showing 40.76 %. As a result, MCF7 cells cultivated in monolayers demonstrated enhanced cellular death, indicating that the IC50 of this medication was roughly 50-fold, 60-fold, and 80-fold higher for spheroids of 300, 400, and 500 m diameter, respectively, when treated with doxorubicin. 61 The nonproliferative cells in the spheroids may affect the effectiveness of drugs such as carboplatin, cisplatin, doxorubicin, oxaliplatin, methotrexate, and paclitaxel in proliferative regions of cells. 62

Imamura et al. reported the expression of Ki67 in 2D cell cultures and spheroids of BT549, BT474, and T47D cells. The results showed that BT549 cells were grown in 2D, showing 84 % of Ki67-positive cells. However, spheroid shows 46.5 % Ki67 expression, indicating that the spheroids had a more significant G0 dormant subpopulation responsible for their resistance to paclitaxel. 63

The three pillars of cancer treatment are surgery, chemotherapy, and radiotherapy. Immunotherapy has emerged as a potential fourth pillar addressing cancer through the immune system's innate mechanisms to distinguish between healthy and diseased tissue. The classical mechanism of the body eradicates the disease on its own by the host immune system. Lately, immunotherapy has been widely used for cancer treatment that gears up the host immune system to destroy cancer and induce a long-term immune response.

## 3. Immunotherapeutic model

Immunotherapy is a powerful and potential tool in cancer treatment. However, patients' response to immunotherapy varies depending on the type of tumor and its microenvironment. 64 The architecture and cellular components of the tumor microenvironment help create resistance to immune cells. In addition, there is the recruitment of immunosuppressive cells and the expression of immune checkpoint inhibitors such as Programmed Death Ligand-1 (PD-L1). 65 The tumor mainly affects metabolism, vascularisation, and the immune system in the tumor microenvironment. Moreover, it modifies tissue-resident immune cells into a tumor-promoting profile; thus, immune cells promote tumor growth and proliferation. 66

The TME enhances the recruitment of myeloid-derived suppressive cells (MDSCs), regulatory T-cells, and increased checkpoint inhibitors such as PD-1 on immune cells, along with the expression of CD47 that avoids phagocytosis of tumor cells. 67 The surrounding fibroblast cells promote tumor growth by promoting tumor-associated M2 macrophages, limiting the entry of T cells toward the tumor. 68 Thus, understanding the complexity of the tumor microenvironment is essential to analyze patients' responses to immunotherapy.

Immunotherapy can be performed in multiple ways to increase the effectiveness of immune response and recognition of tumors by immune cells. Here, the role of cancer vaccines, interferons, cytokines, macrophages, CAR-T cell therapy, and Immune checkpoint blockade in immunotherapy is discussed.

### 3.1. Cancer vaccines

Cancer vaccines are designed to generate the effector T-cell, which can recognize the tumor cells and prevent further tumor progression. The basic principle of cancer vaccines is to enhance the ability of T-cells against tumor antigens toward tumor recognition and eradication. The effect of cancer vaccines depends on the nature, the antigen type, and the antigen's ability to boost the immune response. 69 The tumor antigen should fulfill fundamental criteria to induce the immune response against the tumor; antigen expression should be abundant on the tumor cell but not on the normal cell. Antigen should be immunogenic enough to induce the immune response and must be involved in tumor progression. However, only a few or no tumor antigens fulfill all the criteria. 70

The first effective tumor antigens (TAs) recognized by T lymphocytes were discovered in 1991. TAs were found on various solid tumors 71, such as cancer-germline antigens, mutant antigens, over-expressed antigens, and viral antigens. TAs activate the cellular immune response in cancer patients 72 by generating the epitopes. These are displayed on the surface of tumor cells to major histocompatibility complex (MHC) class I molecules and may stimulate CD8 T lymphocytes. It has been demonstrated that antigen-specific immune responses induced by peptide vaccinations targeting tumor-associated antigens increase cancer patient survival. In phase II clinical trials, immunization with the hCG peptide promoted the development of anti-hCG antibodies in 56 of 77 cancer patients. 73 The recombinant viruses produce tumor-associated antigens, as viruses are inherently immunogenic. 74 In several clinical trials, the carcinoembryonic antigen (CEA) generated T-cell response was observed, along with disease stabilization in up to 40% of metastatic cancer patients. 75 Similarly, a phase II clinical trial examining the effectiveness of chemotherapy in conjunction with non-replicating canarypox virus vaccination with T-cell costimulatory molecule B7.1 showed that 50% of patients exhibited anti-CEA-specific T-cell responses. 76

### 3.2. Cytokines and interferons in immunotherapy

Cytokines are proteins naturally secreted by immune cells for communication. 77 It enhances the efficacy of T-cells when delivered in high doses to metastatic cancer patients. Therefore, it is called immunostimulatory cytokine. 78 Interleukin-2 was approved for treating metastatic kidney cancer in 1991 and metastatic melanoma in 1998 by the US Food and Drug Administration (FDA). 79 IL-2 generates autologous tumor-infiltrating lymphocytes (TIL) and lymphocytes expressing transgenic TCRs to promote survival after adoptive transfer into cancer patients. 80 It may also stimulate Treg proliferation and produce severe toxicity, such as vascular leak syndrome (VLS), pulmonary edema, hypotension, and cardiovascular toxicity. 81 Cytokines directly activate immune effector cells and stromal cells in the primary tumor and enhance the recognition of tumor cells. Several studies have demonstrated that cytokines have antitumor activity in numerous cytokine-based cancer treatments. Several cytokines, including GM-CSF, IL-7, IL-12, IL-15, IL-18, and IL-21, have entered clinical trials to treat advanced cancer. In addition, cytokines have been combined with adoptive cell therapy to develop anti-tumor T-cells.

To date, two cytokines have received FDA clearance as single medicines for cancer treatment. High-dose IL-2 is used for stage III melanoma adjuvant therapy for metastatic melanoma, renal cell carcinoma, and interferon-α (IFN-α). However, the extensive pleiotropism and redundancy of cytokine signaling and the dual function of many cytokines in immune activation and suppression pose challenges to achieving significant anti-tumor responses. 82 Miyashita et al. engineered genetically modified induced pluripotent stem cells to express IFNs against human melanoma cells in xenograft models for immunotherapy. The iPS-ML describing type-I IFNs reduced SK-MEL28 melanoma growth. 83 Ahmadzadeh et al. analyzed the effect of activated regulatory T-cells on IL-2-mediated anti-cancer immune responses. The inhibition of anti-tumor response to IL-2 therapy in melanoma and renal cell carcinoma patients occurs due to the proliferation of regulatory T-cells in humans. However, decreasing regulatory T-cells may improve the ability of IL-2 to stimulate anti-tumor immune responses in cancer patients. 84 Krie et al. investigated the impact of high-dose IL-2 administration on the growth of effector immune cells, such as CD8 T-cells and natural killer cells, in B16 lung melanoma in mice models. It activates the effector immune cells that provide an anti-tumor immunological response. 85

### 3.3. Role of macrophages in immunotherapy

Macrophages are immune cells that perform a wide range of functions, including pathogen eradication, cellular debris removal, tissue growth, homeostasis, and controlling inflammatory reactions. 86 Due to the composition of the cytokine milieu and the surrounding tissue niche, the macrophage may exist in a wide variety of phenotypic states. 87 The activation of macrophages is complex, with two activation states: M1 that is classically activated macrophages or M2 as alternatively activated macrophages. 88 The exposure of lipopolysaccharide, granulocyte monocyte colony-stimulating factor (GMCSF), interferon-γ, tumor necrosis factor (TNF-α), and other pathogen-associated molecular patterns causes polarization of M1 macrophage. 89 M1 macrophages upregulate the genes involved in antigen presentation to improve T-cell responses. 90 M2 macrophages play a crucial role in supporting tumor growth and polarize in the presence of monocyte colony-stimulating factor (MCSF), IL-4, IL-10, IL-13, TGF-β, and glucocorticoids. 91 However, M2 macrophages are essential in normal immune function and homeostasis, such as boosting Th2 responses, removing parasites, immunoregulation, wound healing, and tissue regeneration. 92 Tumors recruit tissue-resident macrophages to the TME and polarize them to an M2 phenotype, thus promoting tumor growth, genetic instability, angiogenesis, fibrosis, immunosuppression, lymphocyte exclusion, invasion, and metastasis. TAMs may create an inflammatory milieu by secreting cytokines, including IL-17 and IL-23, which cause genetic instability. 93 Therefore, a primary goal of macrophage-based cancer therapy is to decrease anti-inflammatory macrophages. Different approaches have been developed to counteract the impact of macrophages, including lowering the amount of TAMs and altering the function of macrophages within the TME.

Limiting the number of TAMs in TME may be achieved by minimizing the existing or preventing the recruitment of macrophages. Blocking the CSF1/CSF1R ligand-receptor pairing for the differentiation and survival of macrophages is a well-established technique for lowering TAM survival. 94 This strategy minimizes the number of TAMs by inhibiting monocyte differentiation and inducing repolarization of TAMs from an M2 toward an M1 phenotype. 95 It also increases tumor sensitivity to other immunotherapies, such as PD-L1 blocking antibodies 96. However, CSF1/CSF1R blockades are not uniformly effective, as they may be balanced by boosting signaling via other prosurvival pathways 97 or by increasing Treg activity in the TME. 98 Altering the function of macrophages within the TME is focused on educating the macrophages that are natural traffic to the tumor and alter the TME to enhance anti-tumor immune response. In this approach, the Andreesen group of Germany in the late 1980s collected monocytes via leukapheresis and cultured them with autologous serum for 7 days to allow differentiation into macrophages. The macrophages were trained with IFN-γ to produce the M1 phenotype and then injected intravenously or intraperitoneally with up to 1.7Χ10^9^ cells per injection. Despite the absence of significant regression at the tumor site, several patients had stable diseased conditions for up to six months after treatment. Disappearance of ascites was observed in two of the seven peritoneal carcinomatosis patients who received intraperitoneal macrophages with increased serum IL-6 in 7 of 15 patients, with no side effects. 99 Ritchie and coworkers showed that in-oxine radiolabeling of educated macrophages actively migrates to sites of metastasis in patients with metastatic ovarian carcinoma, and trafficking occurs for intravenous and intraperitoneal injections. However, the administration of the macrophages appears safe, with no reported high-grade toxicities. 100

Choo et al. have produced M1 macrophage-derived exosome nanovesicles to repolarize M2 TAMs into M1 macrophage in vitro and in vivo. M1 macrophage treatment successfully polarized M2 macrophages to M1 macrophages. 101 Cao et al. developed Ginseng-derived nanoparticles (GDNPs), a potent immunomodulator that participates in mammalian immune response and may represent a novel class of nano-drugs in cancer immunotherapy. It induces the repolarization of M2 TAM in vitro and in vivo, primarily dependent on TLR4 and MyD88 signaling. 102 Li et al. established a 3D model to mimic the tumor microenvironment of human tumor cells, TAMs, and T lymphocytes specific for tumor antigens. The developed model may be used to understand the TAM-mediated reduction of antitumor reactivity and to research TAM regulation in T-cell-based cancer treatment to investigate a PD-1/PD-L1 blocking treatment. 103

Madsen et al. developed multi-cellular tumor spheroids consisting of cancer cells, fibroblasts, and macrophages to study the inhibition and reprogramming of TAM initiated by TAM inhibiting compounds, that is, CCL2 Ab, CSF1R inhibitor, CSF1R Ab, poly I: C, CD40 Ab, and CD40 ligand for their polarization. In 3D MCTS cultures, the polarization of macrophages was assessed by the expression of CD206, CD163, CD86, MHC-II, CD40, CD14, and 43 soluble factors. An inhibitor of CSF1R reduces the infiltration of monocytes into pancreatic cancer spheroids, and macrophages treated with the inhibitor express fewer M2 markers. 104 Klichinsky et al. produced genetically modified human macrophages with CARs to enhance the phagocytosis of cancer cells. They showed that a chimeric adenoviral vector overcame primary human macrophages' intrinsic resistance and produced a proinflammatory (M1) phenotype. CAR macrophages displayed antigen-specific phagocytosis and tumor clearance in vitro. 105

### 3.4. Adoptive T-cell therapy

Cancer immunotherapy employing T cells has long been of theoretical interest. Adoptive immunity has various favorable qualities that make it suitable for cancer treatment: 1) T cell responses are specific and can thus potentially distinguish between healthy and cancerous tissue; 2) T cell responses are robust, undergoing up to 1,000-fold clonal expansion after activation; 3) T cell responses can traffic to the site of antigen, implying a mechanism for eradication of distant metastases; and 4) T cell responses have memory, maintaining therapeutic effect for many years after initial treatment.

Adoptive T-cell therapy is based on the ex vivo selection of tumor-recognizing T cells and their multiplication to attain the target-specific immune response against tumors. In this process, the patient-derived T cells can be modified ex-vivo and then reinfused into the patients to induce recognition and eradication of cancer. However, the method of isolation and identification of antigen-specific T cells is tedious and complicated, and a tiny subset of patients respond to this therapy. Therefore, researchers are focusing on the genetic modification of T cells to express the antitumor receptors on the T cell surface. T cells are modified to express specific antigens or chimeric antigen receptors (CAR) precisely. Despite the remarkable response of engineered T cells, most patients show tumor relapse due to low effector function and short-term survival of infused engineered T cells. 69

TRUCT T cells, or fourth-generation CAR T cells, were developed to increase CAR T cell survival and amplification by incorporating a constitutive or inducible expression cassette encoding each type of cytokine generated by the CAR-T cell to regulate the T-cell response 106 Jacob et al. established that the heterogeneous brain tumor organoids that mimicked antigen escape to target EGFRvIII-receptor by CAR T cells to infiltrate and induce tumor cell death could be efficiently used in preclinical CAR T cell assessment. 107 FRIZZLED-targeted CAR NK cells were developed by Schnalzgr et al. against colon cancer organoids and normal gastric tissue organoids, revealing that these CAR NK cells lacked tumor selectivity. These findings suggest that tumor-derived organoids are potentially therapeutically relevant in vitro models that particularly mimic heterogeneous solid tumors. 108 Michie et al. reported that HER2-targeted CAR T cells induce TNF-mediated killing of colon cancer organoids in a perforin-independent manner, indicating that CAR T cell cytotoxicity against TDOs may need different pathways than two-dimensional cell culture. 109

Li et al. developed an adoptive T-cell therapy to target the human epidermal growth factor receptor (EGFR) in non-small cell lung cancer by expressing a chimeric antigen receptor (CAR) utilizing a non-viral piggy Bac transposon technology. It comprises EGFR scFv, a transmembrane region, and intracellular 4-1BB-CD3 signaling regions. The modified CAR-T cells displayed antigen-dependent growth for anticancer activity in vitro and in vivo. 110 Deng et al. constructed a non-viral third-generation NKG2D CAR and transduced it into T-cells to obtain the NKG2D CAR-T cells. In vitro, NKG2D CAR-T cells showed cytotoxicity and higher IL-2 and IFN-γ secretion against human colorectal cancer cells in a dose-dependent manner compared with untransduced T-cells. NKG2D CAR-T cells significantly suppressed tumor growth, reduced tumor sizes, and extended the overall survival of mice in a xenograft model. 111 Dey et al. developed a dynamic-flow-based 3D bioprinted vascular breast tumor model to examine the response of chemotherapy and immunotherapy. The cell-based immune therapy approach is explored by targeting HER2 CAR receptor-modified CD8^+^ T cells. The CAR-T cell recruitment leads to substantial T-cell activation and infiltration to the tumor site, resulting in up to 70% reduction in tumor mass. 112

### 3.5. Immune checkpoint blockade therapy

Immune checkpoint blockade (ICB) therapy is the most effective form of cancer immunotherapy. Immune checkpoint molecules provide an innate, natural mechanism for regulating the amplitude of the immune response. ICB inhibits the interaction between checkpoint molecules on T cells and their ligands on antigen-presenting cells or cancer cells. It is based on blocking the immune checkpoint inhibitors such as CTL4-A and PDL-1 receptors by using anti-CTLA4 and anti-PD1 antibodies to enhance the antitumor response of cytotoxic T cells. CTL4-A is the T cell surface receptor that downregulates the effector T cells upon binding to the CD80/86 on antigen-presenting cells. Similarly, the PD-1 receptor binds to the PDL-1 ligand on tumor cells and inhibits the T cell activity. 113 Therefore, an FDA-approved monoclonal antibody such as Ipilimumab is used against CTLA-4 receptors, which shows significant progress in treating metastatic melanoma. Moreover, two other monoclonal antibodies, Pembrolizumab and Nivolumab, are used against PD-1 receptors. 114

Zhao et al. constructed a biocompatible and acidic environment-responsive CCM-camouflaged mesoporous silica nanoparticle (CMSN) loaded with dacarbazine (DTIC) and PD-1 to achieve anti-tumor efficacy. In vitro cell experiments demonstrated that DTIC-CMSN exhibits an anti-tumor killing efficiency, having a more vital ability to prolong survival due to highly selective tumor killing, activation of tumor-specific T-cells, and regulation of immune-suppressive tumor microenvironment. to promote the apoptosis of tumor cells in vivo. 115 Lee et al. have investigated the role of B7-H3 in tumor immunity in mouse models. B7-H3-deficient animals or mice treated with an antagonistic antibody to B7-H3 showed decreased development of several malignancies-dependent NK and CD8^+^ T-cells. The suppression of B7-H3 stimulates the activity of cytotoxic lymphocytes in mice. Combining B7-H3 and PD-1 inhibitions improved the therapeutic control of advanced cancers. The B7-H3 checkpoint may thus serve as a potential target for cancer immunotherapy. 116

Marella et al. have developed a three-dimensional alginate-based hydrogel as an extracellular milieu to study the impact of three-dimensionality on the biology and immunological aspects of NB cells. In addition, the cytokine boosted the immune checkpoint ligands PD-Ls and B7-H3 expression in 3D alginate spheres. Consequently, 3D alginate-based hydrogels may constitute a therapeutically beneficial cell culture platform for testing the efficiency of personalized therapeutic approaches to optimize the existing and novel immune-based medicines systematically and dependably compared to 2D culture. 117 Saraiva et al. developed a 3D coculture platform for the MDA-MB-231 breast cancer cell line and patient-derived immune cells for personalized immunotherapy. They have observed broad anti-tumor immune cells; this platform might be used for targeted immune-based treatment. 118 Lugand et al. developed renal carcinoma tumor spheroids to study the immune infiltration for immunotherapeutic study. They observed increased spheroid destruction following treatment with PD1 inhibitors and allowed efficient spheroid formation for a simple RCC 3D model that can be infused with immune cells to study immunotherapies. 119

### 3.6. Role of TME in Immunotherapy

The TME is the biological niche wherein tumor cells, fibroblasts, immune cells, signaling chemicals, and extracellular matrix reside (ECM). Numerous studies have indicated that the tumor microenvironment (TME) plays a crucial role in tumor initiation, progression, and recurrence after treatment and regulates the efficacy of tumor response to immunotherapy. Therefore, more excellent knowledge of the effect of TME on the immune response would aid in enhancing the effectiveness of immunotherapy. The production of co-inhibitory molecules, the release of lactate, and the battle between tumor cells and immune cells for nutrition in TME promote immunological tolerance of a tumor. In addition, cancer-associated fibroblasts (CAFs), the primary cellular constituents of solid tumors, induce immunosuppression by inhibiting T-cell activity and extracellular matrix remodeling. CAFs are susceptible to robust glycolysis and release several cytokines and chemokines that induce tumor immunosuppression, including XCL8, IL-6, tumor necrosis factor (TNF), transforming growth factor β (TGF-β), CCL2, and vascular endothelial growth factor (VEGF), as well as co-regulatory molecules B7H1/ B7DC. 120,121

TME is highly influenced by cytokines, chemokines, and metabolites generated from tumor cells, such as TGF-β, interleukin (IL)-10, and CCXL15. Tumor cells suppress the activity of natural killer (NK) cells and cytotoxic T lymphocytes (CTLs), aiding tumor cells in invading immune recognition and tumor killing. Most tumor cells exhibit a high quantity of stem cell factor, which, by interacting with a c-kit (receptor tyrosine kinase), stimulates the migration of mast cells to the tumor site, which limits the antitumor function of NK cells by producing proinflammatory substances. Colony stimulating factor 1 (CSF1) in TME enhances the transformation and differentiation of TAMs and reduces the function and cytokine secretion. 122 Based on cytokines, macrophages are divided into two subgroups: classical activation (M1) and alternative activation (M2). Macrophages are crucial in eliminating tumor cells; nevertheless, several immunosuppressive signals inhibit their action in solid tumors, thus contributing to tumor growth and metastasis. 123,124 Moreover, TAMs suppress immune cell function by expressing multiple receptors or ligands of the inhibitory receptors (PD-L1, PD-L2, B7-1) 125 by secreting IFN-c through the Janus kinase-STAT3 and phosphoinositide 3-kinase-AKT signaling pathways. TAMs increase the expression of PD-L1 in tumors, and when they bind to the PD-L1 and PD-L2, they increase T cell depletion and promote tumor immune evasion.

Regulatory T cells (Tregs) are a subset of CD4^+^ T cells that influence tumor immunotherapy and vaccine activation by CD4, CD25, and FOXP3 expression. However, Treg with TAMs and MDSCs during tumor growth produces inflammatory cytokines in TME that enhance angiogenesis, tumor cell proliferation, invasion, and metastasis. In addition, hypoxic environments in the TME increase Treg abundance by upregulating FOXP3, encouraging tumor growth. 126 Thus, removing Treg cells from the tumor microenvironment or inhibiting their activity is a strategic approach to cancer treatment. In addition, hypoxia in the TME protects cancer cells from immunological attack and suppresses tumor-killing. 127,128 It influences glucose metabolism, angiogenesis, cell proliferation, invasion, and metastasis, promotes the expression of PD-L1 in tumor cells, and suppresses the T-cell response.

Tumors are complex biological entities that cannot be evaluated by PD-L1 expression levels alone; TILs, the mutational burden, and the probability of neoantigen expression in human malignancies affect clinical outcomes. Therefore, it is more important to consider the nature of immunotherapies and their relationship with the TME while considering immunotherapeutic efficacy. Various cell subsets contributing to an immunosuppressive TME are linked with decreased therapeutic effectiveness, suggesting that the TME plays a role in anticancer immunotherapeutic resistance. Higher levels of MDSCs correspond with a suboptimal response to several immunotherapies, such as immune checkpoint inhibition ACT, dendritic cell (DC) 129,130 immunization, etc. The ratio of effector T cells (Eff T cells) to regulatory T cells (Tregs) is correlated with responsiveness to anti-CTLA-4 checkpoint blockade treatment; more Tregs correlated with reduced effectiveness. 131 Additionally, comparable decisions about biomarkers, such as PD-L1 expression, must be made in patients taking anti-PD-1 or anti-PD-L1 treatment. Patients treated with ipilimumab (an anti-CTLA-4 antibody) displayed a 20% sustained response rate over 5-10 years; pembrolizumab (anti-PD-1) obtains an early response rate of 70-80 %, which declines to 33 % at the three years. In contrast, with the anti-PD-1 and anti-CTLA-4 combo, only 61 % objective response was achieved, along with considerable toxicity. 132 The effect of the tumor microenvironment (TME) on therapeutic response is not limited to immunotherapies; it has been shown for various anticancer treatments, such as chemotherapy. 133 Thus, TME significantly influences the efficacy of anticancer therapies, and its evaluation is crucial for developing successful immunotherapies.

Due to the concentric positioning of the peripheral proliferating tumor cells, viable intermediate cells, and necrotic core cells, the outermost layer of the tumor is mainly exposed to drug treatment. Therefore, the core area of the tumor is unexposed to the drug due to low penetration, enhanced interstitial pressure, physical barrier, acidic pH, and hypoxic conditions, creating drug resistance, which promotes the reoccurrence of the tumor. The microfluidics platform offers the continuous flow of the liquid medium, which successfully eliminates the dead cells, exposes the inner core of the tumor to the drug, increases the drug availability to the tumor's periphery, and helps reduce drug resistance and reoccurrence. Thus, enhancing the efficacy of immunotherapy.

Kast et al. developed a hydrogel matrix for the coculture of human PDAC cells, patient-derived CAFs, and peripheral blood mononuclear cells (PBMCs) to simulate the tumor microenvironment. 134 135 Jiang et al. developed a high-throughput cancer-on-a-chip model for evaluating tumor interactions and immune checkpoint inhibition. They have generated distinct spheroids of breast cancer cells (MDA-MB-231) and Jurkat T-cells using 3D printing. The established model permits the reactivation of T-cells via (IL-2). 136 Courau et al. created a therapeutic coculture model of colorectal tumor spheroids and immune cells. After treatment with an anti-MICA/B antibody, the generated spheroids demonstrate activated memory NK cells invading, killing, and disrupting the three-dimensional structure. 137 Sarchen et al. developed a multicellular therapeutic tumor model for pediatric patients in conjunction with NK cells for cell-based immunotherapy. After the migration of NK cell, the spheroid displays cytotoxicity against the tumor. Targeting Bcl-XL or Mcl-1 reduces the size of the spheroids and boosts cytotoxicity. 138

## 4. 3D tumor model

Conventional 2D cell culture techniques have been widely used for drug discovery and preclinical trials due to their simplicity and cost-effectiveness. However, it fails to mimic the *in vivo* tissue environment. Recently, 3D cell cultures have been widely adopted over 2D cell cultures due to increased extracellular matrix (ECM) expression, which aids cell-cell and cell-matrix interaction, cell proliferation, differentiation, and cellular aggregates. Therefore, a 3D cell culture tool has been employed as an alternative platform for in vitro cell-based experiments, incorporating tissue engineering, fabrication technologies, and cell culture studies. 139

Different 3D cell culture techniques, such as multicellular spheroid, hanging drop method, hydrogel, 3D bioprinting, and 3D scaffolds, have been extensively used to mimic the properties of solid tumors. The 3D cell culture models have been classified as scaffold-based and scaffold-free. The scaffold-based technique primarily enhances cell adhesion to the adhering substrate. In the scaffold-free procedure, however, cells do not require adhering substrate. 140 Herein, the different 3D cell culture techniques with specific advantages and limitations are explained.

### 4.1 Scaffold-free 3D model

The scaffold-free technique entails constructing tissue blocks from the single-cell suspension; however, the scaffold-based method, on the other hand, creates complex tissue architectures from multiple cell types. When mammalian cells grow on the scaffold, they produce more extracellular matrix, which allows them to self-organize and self-assemble tissue blocks. 141

#### 4.1.1. Hanging drop method

The hanging drop method uses specialized plates to create multicellular spheroids, which are then loaded with cell suspensions varying from 50 to 1500 cell density and flipped upside down to create a hanging drop. Cells are bound to form cellular aggregates and interact with adjacent cells at the bottom of the drop. It enables the development of oxygen gradients, nutrients, metabolites, and cellular signaling molecules. Furthermore, improved cell-cell and cell-matrix interactions allow the coculture of tumor cells with immune, fibroblast, epithelial, and endothelial cells.

Kuo et al. created a three-dimensional (3D) tissue culture platform using a polydimethylsiloxane-based hanging drop array. The multicellular spheroid offers tissue-based bioassays for drug screening, coculture, and tumor invasion. 142 Zhao et al. created a 3D-printed hanging-drop dripper plate to grow homogenous tumor spheroids for long-term cell culture, drug testing, and in situ tumor migration and invasion. 143 A high-throughput hanging drop technique was developed to generate HepG2 spheroid to assess morphology, viability, cell cycle distribution, protein content, and protein mass. 144 The hanging drop approach developed a 3D cellular breast cancer model using MDA-MB-231 cells. The morphological properties of 3D models indicated compact cellular aggregates with lower cell proliferation and viability and increased lactate dehydrogenase production. 145 A colorectal tumor spheroid was created to distinguish three multicellular spheroidal stages: normoxia, hypoxia, and hypoxia with necrosis. 146

The hanging drop method produces densely packed spheroids due to the spontaneous aggregation of cells; however, the aggregated cells may be affected by space limitations, rigidity, hydrophilicity/hydrophobicity, and forces that contribute to the aggregations, such as gravity, centripetal force, centrifugal force, magnetic force, and shear force. 147 Therefore, it might fail to simulate cell-cell and cell-ECM interactions due to the highly complex microenvironment for cell growth. Moreover, the medium cannot be changed without disturbing the spheroid due to the low volume. 148

Moreover, the scaffold-free approaches are time-consuming, ineffective at forming uniform spheroid, lacking continuous perfusion, and challenging to monitor real-time and in situ analysis. 149 Therefore, microfluidics-based 3D coculture models have been chiefly employed due to their advantages of flexible cell manipulation, long-term cell culture, and accessibility to combine with multiple analytical techniques. 150

### 4.2. Scaffold-based 3D model

Scaffold-based materials comprise pore-forming biomaterials that aid in gaseous exchange and nutrient distribution. Furthermore, it enables improved manipulation of shape, size, and placement of spheroid as well as stiffness and elasticity of the scaffold. It is commonly employed in tissue engineering and therapeutic applications to regenerate bone, cartilage, ligaments, skin, and skeletal muscles. It employs synthetic and biological materials like matrigel, polyurethane, collagen, and gelatin. The scaffold-based technique includes the porous 3D scaffold, hydrogels, 3D bioprinting, and microfluidics-based 3D cell culture. 151,152

#### 4.2.1. Hydrogels

Hydrogels are three-dimensional scaffolds containing intermolecular or interfibrillar cross-linking of a natural or synthetic hydrophilic polymer to generate swelling structures with high water content. They have a network-forming ability to help cellular aggregation and circulate nutrients, oxygen, and growth signals. 153 Hydrogels from naturally occurring materials like collagen, gelatin, fibrin, agarose, hyaluronic acid, and alginate resemble natural tissue and mimic physiological functions such as cell survival, proliferation, and differentiation. 154

Synthetic hydrogels have several benefits, including desirable porosity, customizable stiffness, and ease of functionalization. Moreover, they are inexpensive and inert. Tong et al. developed polyethylene glycol hydrogels with configurable matrix stiffness to provide a biomimetic niche for studying the biological interactions of numerous cell types, including cancer cells and stem cells. 80 Wen et al. designed a hydrogel scaffold with a particular cancer cell-adhesive surface utilizing b-cyclodextrin-based host-guest chemistry to generate multicellular spheroids. 155 Furthermore, Imaninezhad et al. demonstrated a macroporous hydrogel to create multicellular tumor aggregates, demonstrating the importance of cell-matrix interactions. 156 The thermoresponsive poly N-isopropyl acrylamide-based hydrogel microwell array (PHMA) was developed to generate cancer spheroids from cancer and fibroblast cells for disease modeling and drug screening. 157

Pradhan et al. produced a biosynthetic polyethylene glycol-fibrinogen hydrogel to cultivate breast cancer cell lines MCF7, SK-BR-3, and MDA-MB-231, and high cell viability was observed. 158 Lewis et al. created hydrogels to produce hypoxia in tumor spheroids and found that hypoxia-induced hydrogels had higher invasion than non-hypoxia-induced hydrogels. Hypoxia-inducible factor HIF-1α directs sarcoma cell motility and matrix remodeling. Furthermore, cells migrate long distances toward a higher oxygen gradient. 159 Shaibani et al. created a light-addressable potentiometric sensor combined with pH-sensitive hydrogel nanofibers to detect changes in tumor pH caused by lactate release to understand tumor metabolism and therapeutic response. They discovered a pH drop in multidrug-resistant tumors in the presence of doxorubicin; however, there was no change in the control. This method aids in the understanding of cancer cell metabolism and its response to chemotherapy. 160

Naturally derived hydrogel materials such as collagen or matrigel significantly succeed in providing *in vivo* cell culture conditions. However, they are processed from live tissues containing multiple growth factors that may cause batch-to-batch variations and interfere with biological signaling pathway studies. Synthetic polymers have recently been used to fabricate hydrogels to overcome the limitations of natural polymers since they are affordable and produce predictable and consistent outcomes. However, the requirement for biological moieties may be a limitation in recreating natural EMC. 161

#### 4.2.2. 3D Bioprinting

3D bioprinting is a computer-assisted technique for creating functional tissues and organs, customized composite structures, and autologous cells that mimic physiological conditions. 162 3D bioprinting allows the integration of living cells with biomaterials, wherein multiple layers of cells are deposited to maintain the viability of cells in 3D architecture. It accurately deposits various cell types, thus resembling tissue or organs with mass production. 163

The 3D bio-printed spheroid shows high viability, proliferative activity, and efficient tumor-stromal interactions. 164 Zhou et al. used 3D bioprinting technology to create a biomimetic bone matrix to study the interaction of breast cancer cells with bone stroma. The coculture produced more vascular endothelial growth factor (VEGF) than the monoculture. 165 Kim et al. used a 3D bioprinter to create 3D scaffolds from gelatin methacryloyl and bladder cancer cells to examine the secretion of E-cadherin and N-cadherin in tumor spheroids. 166 Ma et al. used a quick light-based 3D bioprinting approach to create a photocrosslinkable decellularized extracellular matrix to generate HepG2 spheroids. It showed reduced growth as well as an increase in invasion markers. 167 Lee et al. developed a fibrin-based 3D printed model to generate glioblastoma spheroids. The generated spheroids show cell viability for 12 days with upregulated levels of cancer and stem cell-associated markers such as CD133 and DCX. 93 A 3D bio-printed tumor model has been developed by Wang et al. wherein the proportion of GSCs and EMT-related genes is significantly increased with improved stemness resulting in higher drug resistance. 168

The bioprinting techniques used in constructing functional tissues require a bio-ink that creates diverse effects on the encapsulated cells. However, the hardening of bio-ink requires photo-polymerization, and the photoinitiators cause cytotoxicity due to the damage inflicted by UV (10-400 nm) or near-UV blue (400-490 nm) irradiation. 169

#### 4.2.3. 3D Scaffold

A 3D scaffold is a commonly used approach for producing 3D cell culture. It supports cell adhesion, proliferation, signaling, migration, and the development of cellular aggregates mechanically and physically. 170 Scaffolds are typically porous or fibrous, made of synthetic polymers such as polyglycolic acid, polylactic acid, polylactic-co-glycolic acid, polycaprolactone, or natural polymers such as collagen, hyaluronic acid, fibrin, alginate, gelatin, silk, and chitosan to mimic the critical features of ECM. Furthermore, synthetic polymers can be modified or integrated with growth factors, hormones, and cell adhesion molecules to promote cell proliferation.

Wang et al. created a 3D permeable chitosan-hyaluronic acid (CS-HA) scaffold to explore glioma spheroids' morphology, gene expression, and tumorigenicity in vitro. They discovered the expression of biomarkers in glioma spheroids compared to traditional 2D cell culture. 171 A stiffness-varying chitosan-hyaluronic acid scaffold was developed to investigate the influence of stiffness on morphology, proliferation, treatment resistance, and gene expression in human glioblastoma cells. Glioblastoma cells cultivated on stiffer scaffolds demonstrate substantial resistance to chemotherapeutic treatment and strong expression of invasion-related markers compared to 2D cell culture. 172. MDA-MB-231 shows higher collagen content and LOX levels than the MCF-7 cell line.

Porous scaffolds can be created using a variety of processes, including particle leaching, emulsion templating, foam-based scaffolds, and fiber scaffolds. Particulate leaching is when the polymer is combined with an organic solvent and porogen, cast into the mold, and allowed to solidify. In the emulsion templating method, the porous scaffold is fabricated using high eternal phase emulsion. The foam-based scaffold is fabricated using the gas-forming technique, wherein high-pressure gases control porosity. Furthermore, fibrous scaffolds provide a higher surface area for cell adhesion and nutrition and gas exchange to mimic cell alignment. The scaffold material, pore size distribution, and pore interconnectivity may influence the activities of developing cells. Therefore, those are critical parameters for the in vitro 3D cell scaffold. 173

Kailei Xu et al. designed a porous chitosan-alginate scaffold for bone tissue engineering and cancer stem cell enrichment. They studied the effect of scaffold stiffness on PCa cells, that is, PC-3, C4-2B, and 22Rv1. The chitosan-alginate scaffold promotes PCa growth and phenotypic expression. 174 Totti et al. developed fibronectin-coated 3D porous polyurethane scaffolds to mimic pancreatic tumor microenvironments. The constructed 3D scaffold demonstrates the generation of dense cellular masses by synthesizing collagen-I and establishing environmental stress gradients. 175 Rijal et al. created a 3D macroporous polycaprolactone scaffold for culturing primary breast cancer cells and cancer-associated fibroblasts (CAFs) to monitor tumor activity and drug response. 176

Due to the porous structures and tunable surface properties, 3D scaffolds possess many advantages over other 3D cell culture techniques in mimicking the in vivo condition. Incorporating a microfluidic platform in 3D scaffolds aids gaseous exchange, creating an oxygen gradient with a continuous supply of nutrients and growth factors resembling 3D architecture like an in vivo tissue environment.

## 5. Microfluidics 3D tumor model

Microfluidics, commonly known as Lab-on-a-chip, is an emerging technology in the pharmaceutical industry, diagnostics, healthcare, and life science research. 177 It provides a vast array of applications in biology since it has been continuously employed for drug discovery and development, toxicity analysis, cell culture, genetic assays, protein studies, intracellular signaling, stem cells, and tissue engineering. 178 The microfluidic system provides the advantage of microscale dimensions, chemical gradients, gaseous exchange, and replacement of a nutrient medium, thus enhancing cell viability. 179 Microfluidic devices can be fabricated using photolithography, thin film deposition, wet hydrofluoric etching, access hole forming, and chip bonding. 180

In 2D cell culture, animal cells or tissue taken from the host are cultivated in multi-well plates or tissue culture flasks by constantly removing waste media and replacing it with a new medium for nutrients and growth factors. However, in vivo conditions necessitate constant blood perfusion with 3D architecture. The absence of 3D architecture profoundly alters cell behavior, such as the absence of cell-cell contact, tissue-specific architecture, and mechanical and biological signals. 181 The 3D cell culture platform has the potential to provide tissue-like architecture with better cell-cell and cell-matrix interaction, helping the cell to receive chemical and mechanical cues from the surrounding environment for cell proliferation and growth. A three-dimensional collagen scaffold implanted with cancer cells and human umbilical endothelial cells was created to generate vessel-like formations and evaluate anti-invasive and anti-metastatic drugs. 182 Endothelial cells and macrophages were cocultured on a microfluidics-based fibrous scaffold, increasing TNF-α production. 183 A diffusion-driven cell-embedded scaffold with simultaneous and orthogonal gradients was designed to mimic the localized differentiation of motor neurons in the neural tube. 184

Though the 3D cell culture model allows cell-cell and cell-matrix interaction, static 3D cell culture techniques fail to provide constant perfusion and mimic physiological conditions. Therefore, adding perfusion with the help of a microfluidics tool creates an oxygen gradient with control over nutrient and growth factor exchange.

### 5.1. Perfused 3D cell culture

Incorporating microfluidic devices with 3D culture helps to address the fundamental questions in cancer biology. Microfluidics-based 3D cell culture techniques provide better accuracy for analyzing various complex biological processes. It supports cell interaction, migration, and parallel multi-chamber designs, permitting the initial compartmentalization of cellular subtypes in adjacent microenvironments, including chemical and cellular communication across chambers via microchannel networks. It can create a complex in vitro environment, allowing the formation of steep molecular gradients, cell chemotactic mechanisms, the recreation of several TME characteristics, and the opportunity to study the interactions between various immune, stromal, and tumor cell types. Therefore, adopting microfluidic models in the pharmaceutical industry plays a significant role in drug development. Thus, microfluidics 3D cell culture models can potentially reduce and replace 2D assays and animal models, providing a scalable and versatile platform for immunotherapy. 185

Perfused 3D cell culture aids in regulating physiological conditions and minimizes fluctuating microenvironments such as pH, oxygen, glucose, and growth factors. It mimics circulation and manages chemostatic requirements by creating an oxygen and growth factor gradient, influencing the secretion of chemical cues such as growth and survival factors, cytokines, morphogenetic proteins, metalloproteinase, death ligands, steroid hormones, peptides, and ions, and maintaining cell-cell and cell-ECM interaction. 186 A microfluidic device was developed to accomplish high-density three-dimensional culturing of adherent cells. It permits quantitative analysis of three-dimensional cultures under dynamic conditions, with implications for stem cells, organs-on-chips, and cancer research. 187

The mimicry of *in vivo* solid tumors demands a heterogeneous tumor microenvironment with a coculture of multiple cell types such as tumors, endothelial, fibroblast, and immune cells. Therefore, the microfluidics-based 3D coculture model helps establish 3D tumor spheroids, heterogeneity, and *in vivo* solid tumor-like architecture. The 3D coculture and continuous perfusion closely resemble the physiological condition by allowing control over cocultures, perfusion flow, and signaling gradient. It involves patterning animal cells with an extracellular matrix environment to create a coculture of multiple cell types with continuous medium perfusion, basal access, and gradient formation. 188 Further, it provides the gradient of soluble biomolecules in different biological events like angiogenesis, tumor invasion, and migration. 189 Moreover, the micro-dimensions allow low consumption of expensive cell material and reagents. The reliable thinness due to the well-defined height of microchannels, improving the imaging quality and speed, makes the microfluidics platform ideal for perfusion-enabled in-vitro 3D cell coculture. 190

In conjunction with a porous 3D scaffold with coculture, a microfluidics platform serves as a standalone system that helps recapitulate *in vivo* tumor microenvironment for disease modeling, understanding, discovery, and high-throughput drug screening. A microfluidic-based 3D tumor spheroid was developed by Chen et al. to mimic the invasive tumor microenvironment by recapitulating epithelial-mesenchymal transition. 191 A self-organizing microfluidic system was developed to generate a multicellular spheroid composed of cancer cells, vascular endothelial cells, and a type I collagen matrix to mimic the tumor microenvironment. 192 Aung et al. developed a perfusion-based tumor on-chip model to coculture cancer cells, monocytes, and endothelial cells to evaluate the tumor-macrophage interaction. 193

Microfluidics and perfusion efficiently simulate the blood flow and thus take the artificial 3D scaffold model (spheroids) closer to mimicking the in vivo condition. Further, the perfusion model coupled with nanotechnology-aided molecular recognition and targeted drug delivery can enhance the efficacy of the immunotherapy.

### 5.2. siRNA-nano immunotherapy

The small interfering RNA (siRNA) mediated gene silencing technique has gained significant attention due to its target specificity. Subsequently, tumor regression can be applied individually or as a combination therapy. 194 siRNAs are small double-stranded nucleotides that bind to the complementary mRNA, mediate RNA cleavage, and block protein synthesis. Despite the abundant potential, low stability, and shorter life span of siRNA during delivery to the target gene, it degrades before reaching the target gene. Therefore, finding an appropriate delivery vehicle for effective siRNA delivery is imperative. 195 Nanomaterials have the enormous potential to carry and deliver the siRNA to its specific target. It has been widely used as a targeted delivery platform for practical therapeutic approaches. Inorganic nanomaterials such as quantum dots, gold, silica, and magnetic nanoparticles and organic nanomaterials such as liposomes, lipids, dendrimers, and micelles have been utilized. 196,197 Thus, the nanoparticle-mediated siRNA delivery platform can be used in cancer treatment. This approach helps in silencing CTLA-4 and PD-1 genes, resulting in the upregulation of CD8 T cells by creating a pro-immune modulatory tumor environment, gearing the ability of T cells towards tumor recognition. siRNA nanomaterials and other chemotherapeutic drugs can be applied to decrease the tumor burden.

Ferritin nanocage containing 24 PD-L1 binding peptides was constructed for specific binding of PD-L1 expressing tumor in coculture of cancer cells and T cells. The developed construct inhibits the PD-1/PD-L1 interaction, restores the T cell activity, and shows antitumor activity. 198 Lactic-co-glycolic acid nanoparticles were synthesized for a pH-responsive co-delivery of immuno-metabolic modulator metformin and siRNA targeting fibrinogen-like protein mRNA. It promotes T-cell-mediated antitumor immune response and enhances antitumor immunity. 62

A spheroid-based 3D coculture of human endothelial cells, immune cells, fibroblasts, and ccRCC cell lines was established by Rausch et al. to mimic clear cell renal cell carcinoma. They observed the secretion of ECM and immune cell infiltration in spheroid co-cultures within six hours, along with PDL-1 expression. 199 Cess et al. developed a 3D tumor microenvironment of human tumor cells, tumor-associated macrophages, and tumor antigen-specific T cells to study TAM modulation of T-cell-based cancer immunotherapy. The developed tumor microenvironment-mimicry helps to learn the TAM-mediated suppression of T-cell antitumor reactivity. 200

A 3D coculture of multipotent mesenchymal stromal cells and their osteogenic derivatives with endothelial progenitor cells was developed to mimic bone marrow niche and study engineered immune cells' effect on primary myeloma cells. 201 A 3D coculture model of pancreatic cancer cells, CAFs, and monocytes was established to identify the cellular mechanisms that induce an immunosuppressive tumor microenvironment. They have observed fibroblast-induced production of immunosuppressive cytokines that promote the polarization of M2-like macrophages and myeloid-derived suppressive cells (MDSCs) and inhibit CD4^+^ and CD8^+^ T cell activation. 202 A 3D alginate-based hydrogel was prepared to analyze the IFN-γ-induced expression of surface molecules on NB cells capable of tuning the antitumor activity of NK cells. They have observed that IFN-γ induces the face of high amounts of HLA-I molecules, which protected NB cells from the attack mediated by KIR/KIR-L matched NK cells. Moreover, the cytokine increased the expression of the immune checkpoint ligands PD-Ls and B7-H3. 203

A scaffold-free 3D protocol was developed to coculture MDA-MB-231 breast cancer cells and patient-derived immune cells to study the crosstalk between both cell types and assess personalized therapeutic approaches to identify the antitumor immune response. 204 Al-Samadi et al. developed a 3D microfluidic chip to coculture the (HSC-3) tongue cancer cell line embedded in a human tumor- Myogel/fibrin to test the efficacy of immunotherapy. 205 A 3D multicellular spheroid model was developed to coculture the cancer cells, fibroblasts, and monocytes to analyze the infiltration and differentiation of monocytes in spheroids. They have observed high CD206 and CD14 expressions on infiltrating monocytes to M2-like macrophages. 206 A cytocompatible chitosan-based thermal was prepared to encapsulate viable CD8 T lymphocytes with tumor cells. CD8 T lymphocytes encapsulated in this formulation retain their anticancer functions. 207 An immunoreactive organoid composed of murine 4T1 cells with activated splenocytes in extracellular matrix hydrogels was prepared to study the effect of *in vitro* immune checkpoint blockade therapy. 208 Cui et al. developed an immunosuppressive glioblastoma (GBM) microenvironment in a microfluidic'-based ex vivo system that is 'GBM-on-a-Chip to study allogeneic CD8^+^ T-cells trafficking through 3D brain microvessels and infiltrating brain-mimicking tissue. 209 Alginate hydrogel tubes were prepared to create a cell-friendly microenvironment for growing primary human T cells for adoptive immunotherapy. 210

## 6. Summary and future perspective

The immune system is integral to health as it promotes wellness, prevents illness, and fights against disease. An essential goal of immunotherapy is to help the immune system recognize cancer cells as non-self instead of self. Immunotherapy is a potential new cancer treatment as it can turn the immune system's power— more powerful than any cancer drug —against cancer cells. In the future, immunotherapy will be regarded as a fourth modality in traditional cancer treatment, along with surgery, radiation therapy, and chemotherapy. Immunotherapy is often administered with conventional cancer therapies and occasionally as the primary therapeutic modality. 211

However, the process of tumor growth and the role of TME in tumor progression must be thoroughly investigated. The TME is an essential part of cancer initiation and progression. However, knowledge of mechanisms involved in the development of TME and disease progression is in its infancy. Emerging data showed that an intricate understanding of the TME is crucial to identifying predictive biomarkers of response that can routinely be used in the clinic. 212

3D cell cultures permit intra-tumor and tumor—stroma contacts, thus, closely resembling an accurate tumor mass. A 3D cell culture could help research cell adhesion, motility, and cell-cell and cell-matrix interactions, which are challenging to study in animal models. Moreover, in a 3D setup, cancer cells are exposed non-homogeneously to nutrients and oxygen, and the neoplastic cells receive a differential energy supply. Similarly, drugs may not be able to penetrate the entire 3D cell culture, ensuring the data's reliability, meaning accuracy in predicting the antitumor activity of bioactive molecules. It effectively mimics the natural tissue environment through coculture, which allows multiple cell types to be cultured to produce tumor spheroids. 20

Furthermore, the microfluidic-based 3D cell culture platform with perfusion provides a regulated environment for cellular architecture, ECM, cytokines, and oxygen levels. The laminar flow, size of the microchannels, and volume ratio of the cell to extracellular fluid are very similar to those in the TME, creating a concentration gradient identical to that in vivo. The controlled fluidic motion mimics various mechanical signals, including shear stress and physiological flow, such as blood flow, and tissue-specific motions, such as cardiac rhythms and respiratory. Finally, 3D technologies offer increased stability and longer lifespans, suitable for lengthier experiments. 17

Furthermore, tumor spheroids exhibit chemokine gradients, cell contact, migration and adhesion, hypoxia, and gaseous exchange, indicating a more relevant tumor microenvironment. As a result, it can be used to investigate various topics, including how the tumor microenvironment promotes cancer progression, the metastatic cascade, and how the tumor spreads, all of which are linked to angiogenesis, migration, intravasation, and extravasation. 21 It may aid in developing tailored therapy, such as immunotherapy and high throughput drug screening, bringing it one step closer to the in vivo situation. Another benefit of adopting a microfluidic platform is that it can be employed in preclinical experiments, reducing the ethical and financial load. However, the process of tumor growth and the role of TME in tumor progression must be thoroughly investigated. As a result, the microfluidic-based 3D coculture system could eventually replace current in vitro and in vivo models. As a result, we are expanding our understanding of mechanisms and building predictive and efficient techniques for cost-effectively designing immunotherapies.

Furthermore, siRNA nanoparticle-mediated gene therapy combined with a 3D cell culture technology is a powerful tool for generating customized medicine and high-throughput drug screening. However, more research is needed to create a perfusion-enabled 3D scaffold platform for testing the efficacy of in vitro immunotherapy.

The effect of *in vitro* immunotherapy has been tested using different 3D cell culture methods. However, a microfluidics-based 3D cell culture platform is an emerging technology that provides continuous perfusion to closely mimic physiological conditions one step closer to the *in vivo* condition.

## 7. Conclusion

This paper discusses 3D cell culture models utilized as immunotherapeutic platforms, each with its benefits and drawbacks. However, researchers are concentrating on more realistic in vivo properties as an immunotherapeutic model using a perfusion-enabled microfluidic-based 3D cell coculture platform. Targeted nanotherapeutics coupled with immunotherapy will change the course of cancer therapy. The understanding gained will be more effective and reduce side effects, increasing patient compliance.

## Figures and Tables

**Figure 1:**
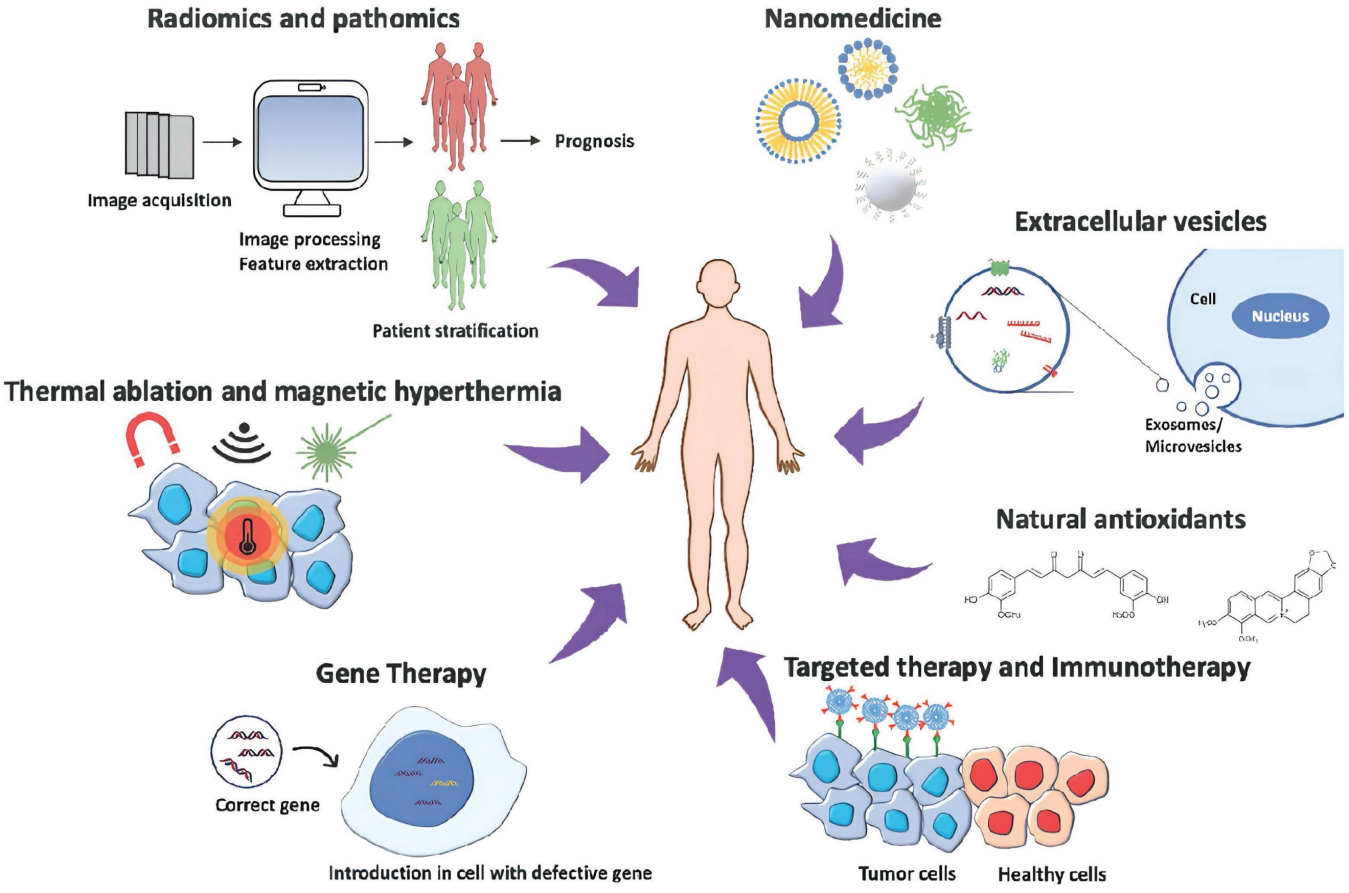
Cancer therapy approaches: The image represents the most innovative strategies to treat cancer, combining disciplines to obtain the most efficient and personalized patient treatment. The illustration is adapted from ref. 17.

**Figure 3:**
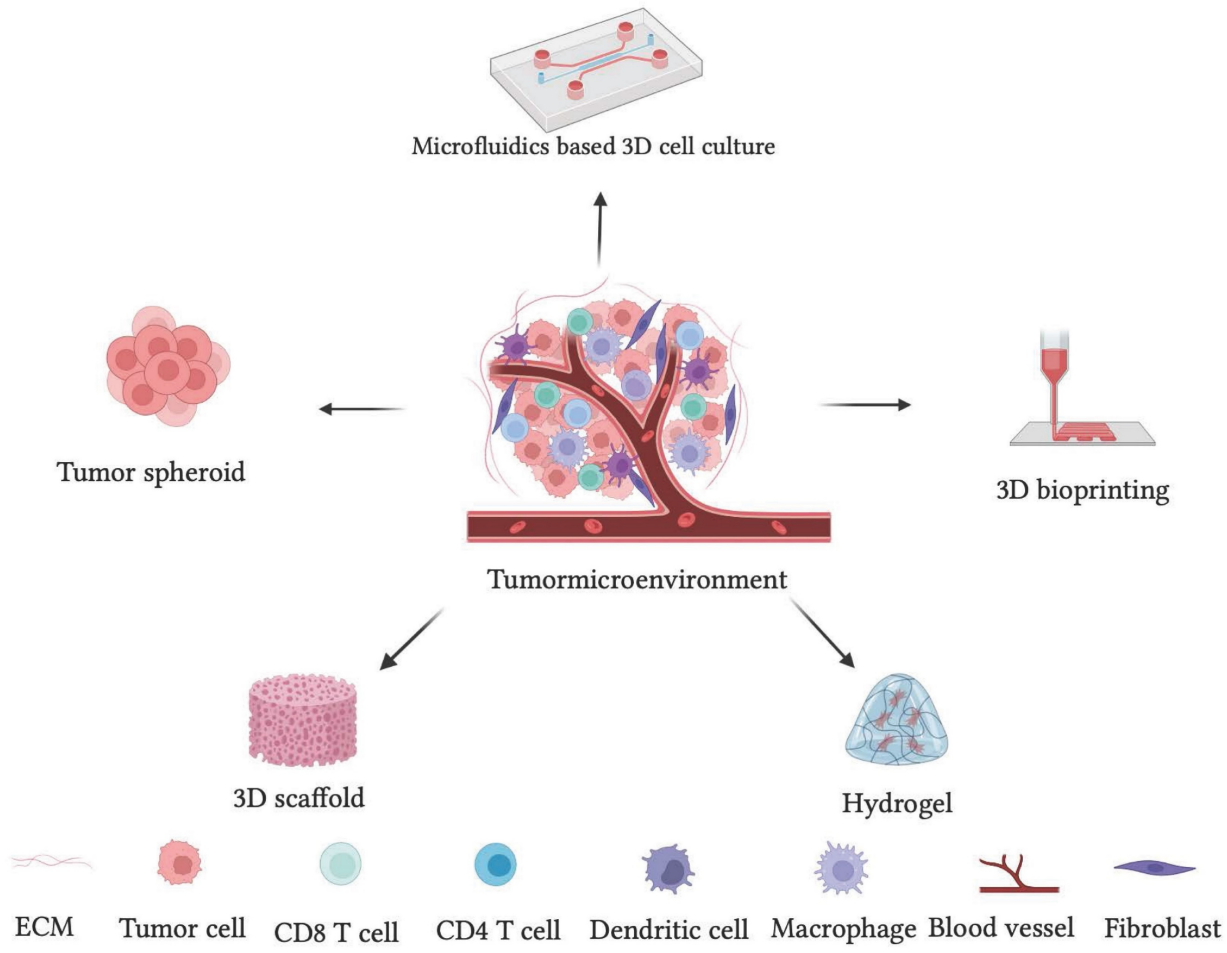
Schematic representation of different 3D cell culture techniques used to recapitulate the complexity of tumor microenvironment as a disease model.

**Figure 4:**
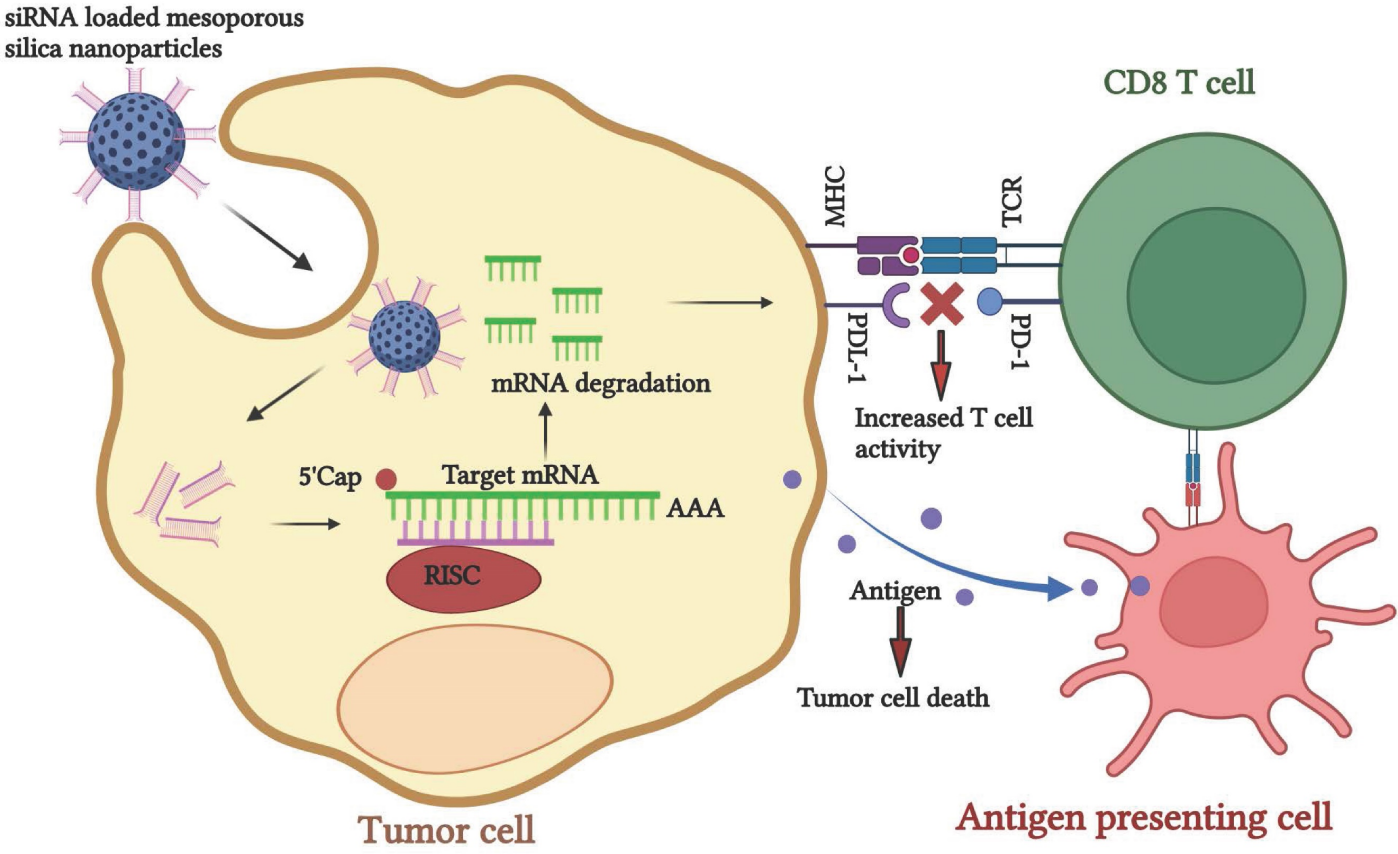
Schematic representation of siRNA-nano therapeutics for knockdown of PDL-1 to enhance the tumor recognition activity of CD8 T cells for cancer immunotherapy.
